# Concurrent Vulvar Vitiligo and Lichen Sclerosus: The Importance of Melanocyte-Specific Immunostains in Resolving Diagnostic Ambiguity

**DOI:** 10.7759/cureus.104909

**Published:** 2026-03-09

**Authors:** Nabor S Mireles, Kaitlyn Levett, Annie Dai, Abdul Hafeez Diwan, Omid Jalali

**Affiliations:** 1 School of Medicine, Baylor College of Medicine, Houston, USA; 2 Department of Pathology and Immunology, Baylor College of Medicine, Houston, USA; 3 Department of Dermatology, Baylor College of Medicine, Houston, USA

**Keywords:** autoimmune dermatopathology, lichen sclerosus, melan-a immunostain, vitiligo, vulvar dermatoses

## Abstract

Vitiligo and lichen sclerosus (LS) are autoimmune dermatoses that may share overlapping clinical and histopathologic features, posing diagnostic challenges when they occur concurrently. We report a biopsy-confirmed case of vulvar vitiligo and LS in a 61-year-old woman presenting with pruritic, depigmented patches on the labia majora and minora. Histopathologic examination showed epidermal thinning with papillary dermal hyalinization and a dense, band-like lymphocytic infiltrate consistent with LS. Melan-A immunostaining demonstrated a complete absence of epidermal melanocytes, confirming concurrent vitiligo rather than LS-related post-inflammatory hypopigmentation. This case underscores the importance of correlating subtle clinical clues with melanocyte-specific immunostains to distinguish coexistent vitiligo and LS, enabling accurate diagnosis, appropriate management, and reduction of long-term risks such as scarring or malignant transformation.

## Introduction

Vitiligo is an acquired autoimmune disorder characterized by selective epidermal melanocyte destruction, clinically manifesting as sharply demarcated, depigmented macules and patches. Histologically, vitiligo demonstrates a complete absence of epidermal melanocytes without significant epidermal or dermal inflammatory changes [1]. Lichen sclerosus (LS) is similarly suspected to be autoimmune and typically presents as hypopigmented, atrophic plaques predominantly affecting anogenital regions. On histopathology, LS reveals epidermal thinning, basal layer vacuolar alteration, pronounced dermal collagen hyalinization, and dense lymphocytic infiltration [2]. Over time, LS can lead to fissuring, scarring, and architectural distortion and is associated with an increased risk of vulvar squamous cell carcinoma [2]. These potential sequelae underscore the importance of early diagnosis, treatment, and longitudinal monitoring. Here, we report a case of concurrent vulvar vitiligo and LS, highlighting the diagnostic overlap and the value of histopathology and melanocyte-specific immunostaining in confirming true melanocyte loss and guiding management.

## Case presentation

A 61-year-old postmenopausal woman was referred by gynecology for evaluation of persistent vulvar pruritus and a progressive depigmented rash unresponsive to several over-the-counter moisturizers. The clinical differential diagnosis included vulvar vitiligo and early LS. She characterized the rash as intensely pruritic, refractory to topical self-care, and gradually worsening over approximately one year. She denied any personal or family history of autoimmune disorders.

Physical examination revealed symmetric, well-demarcated depigmented patches involving the bilateral inguinal folds, labia majora, and labia minora without evident atrophy. Although initially suggestive of vulvar vitiligo, subtle reflective surface texture and prominent pruritus heightened clinical suspicion for underlying LS. Given the clinical ambiguity, a 4 mm punch biopsy was performed on the right labium majus for histopathologic evaluation.

**Figure 1:**
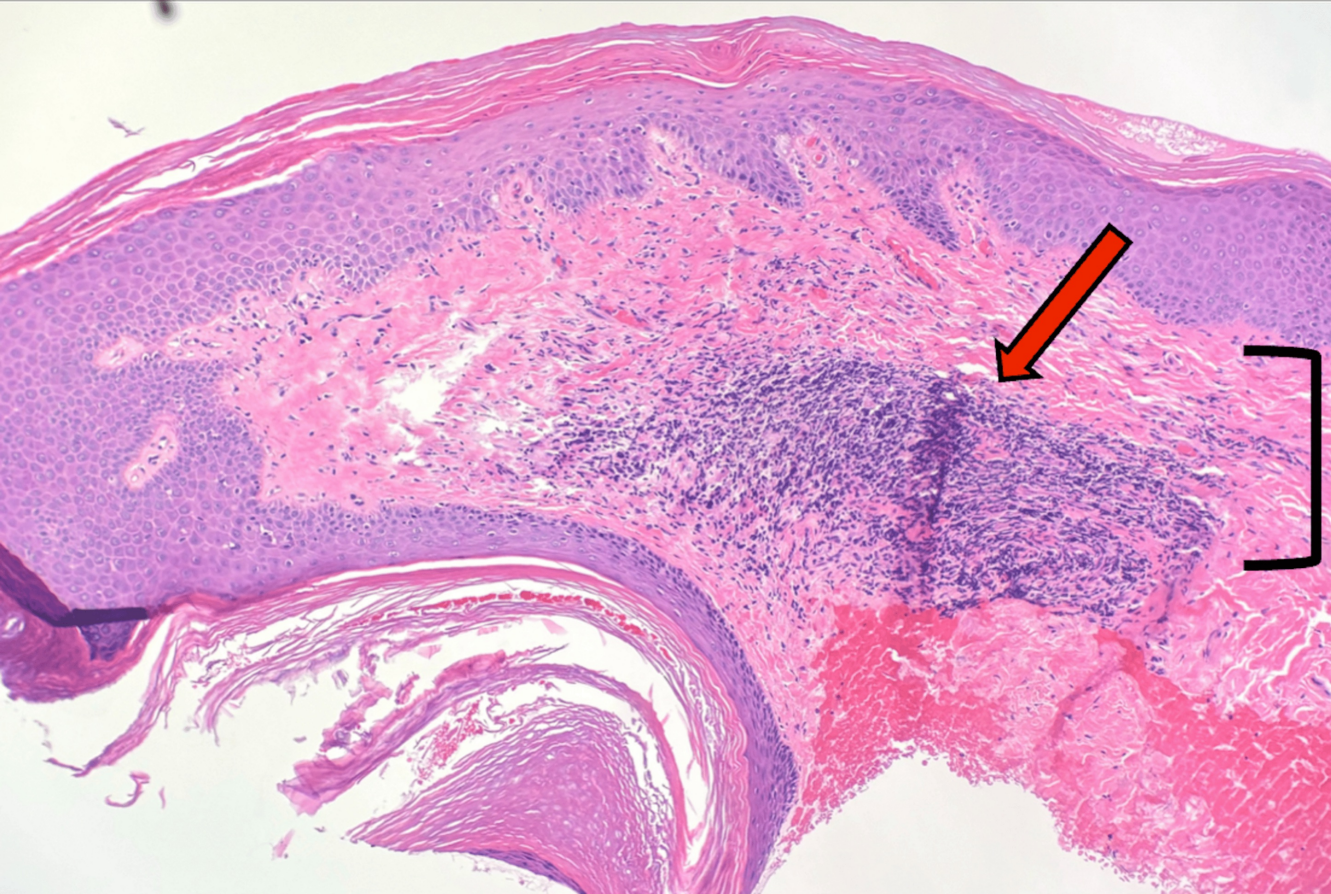
Punch biopsy (H&E, 20×) A dense lymphocytic infiltrate (red arrow) is present in the superficial dermis (black bracket).

**Figure 2:**
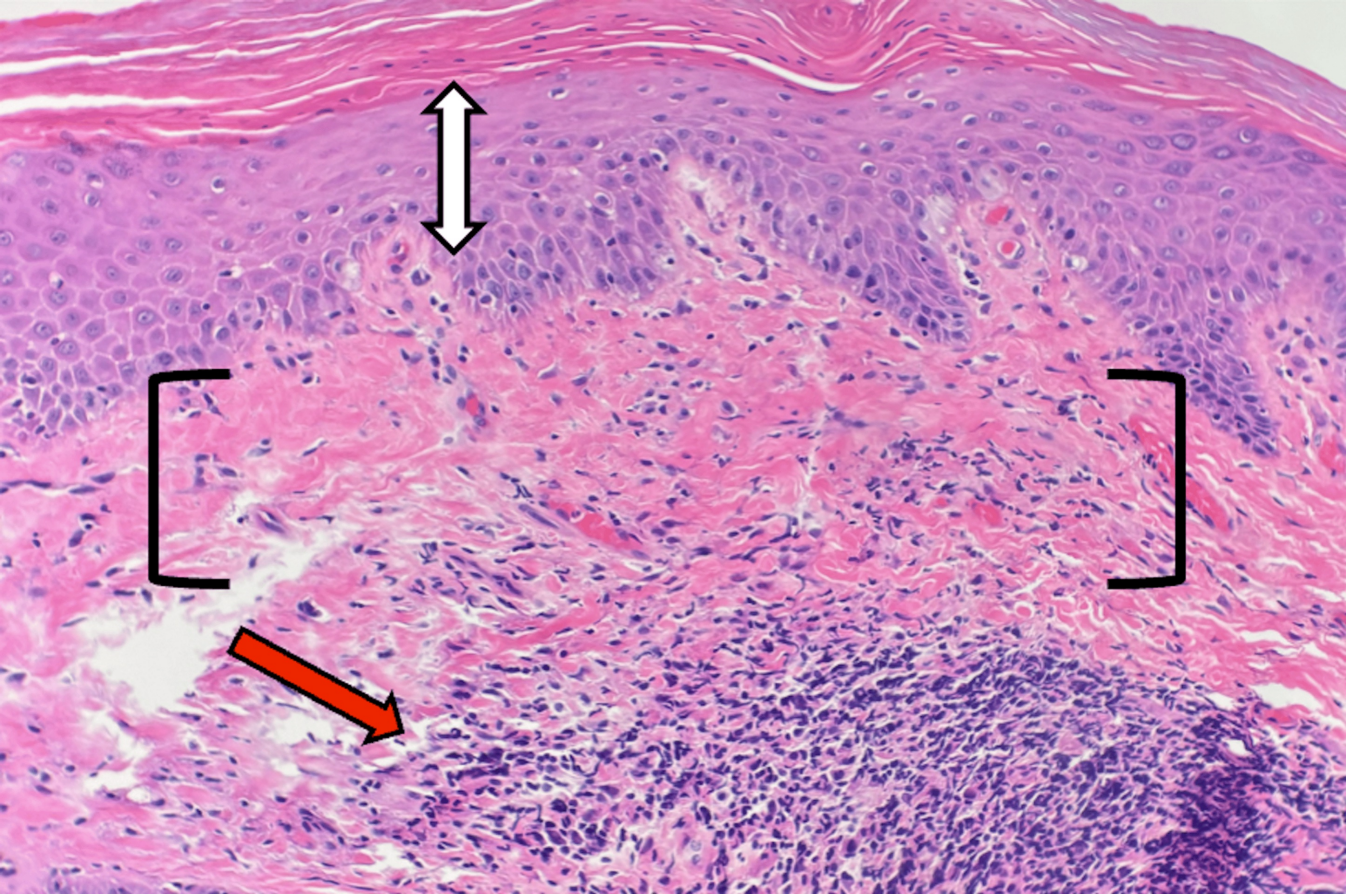
Permanent section (H&E, 100×) Mild epidermal thinning (white double-headed arrow) is present over a homogeneously hyalinized papillary dermis (black brackets) and a dense, band-like lymphocytic infiltrate (red arrow).

**Figure 3:**
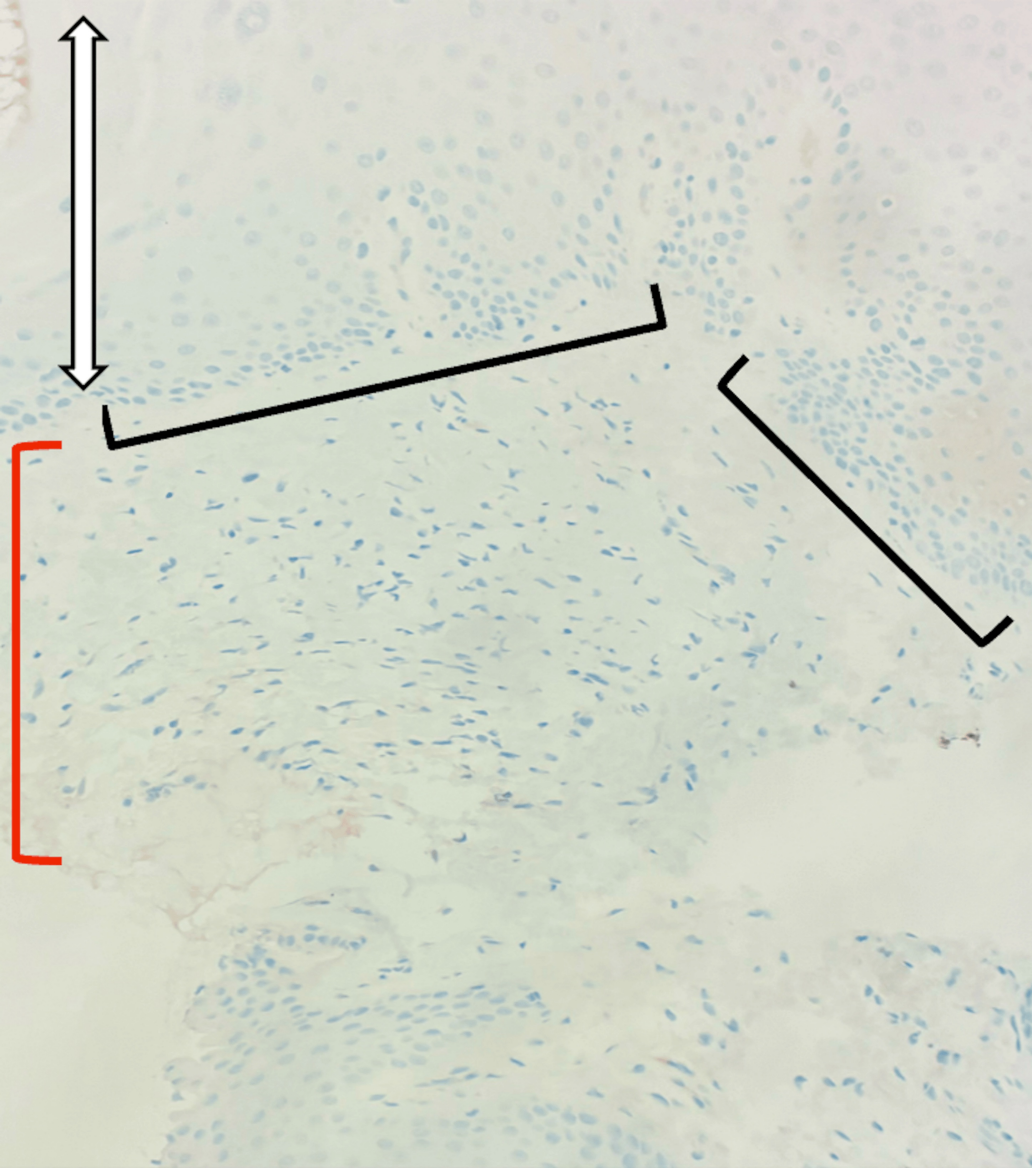
Permanent section (Melan-A immunostain) A complete absence of epidermal junctional melanocytes is demonstrated by the lack of brown Melan-A immunostaining along the dermal-epidermal junction (black brackets), consistent with complete epidermal melanocyte loss. The epidermis is indicated by the double-headed white arrows, and the dermis is labeled by the red bracket.

Histologic examination demonstrated characteristic LS findings, including a homogeneously hyalinized papillary dermis and a dense, band-like superficial dermal lymphocytic infiltrate (Figure 1, Figure 2). Melan-A immunohistochemical staining confirmed the complete absence of epidermal melanocytes, strongly supporting a concurrent diagnosis of vitiligo (Figure 3). The patient was therefore started on a structured topical regimen with betamethasone 0.05% ointment, applied twice daily for four weeks, then once daily for four additional weeks, for a total taper duration of eight weeks. She was also scheduled for regular gynecologic follow-up to monitor disease progression and to surveil for the malignancy risk associated with vulvar LS. At the time of manuscript preparation, the patient had not returned for formal dermatology reassessment and was considered lost to follow-up.

## Discussion

Our patient’s presentation adds to the limited adult literature describing coexistent vulvar vitiligo and LS, confirmed clinically and histopathologically [3]. Initially, clinical differentiation was challenging due to pronounced depigmentation lacking obvious atrophy. Published overlap descriptions often emphasize more overt LS morphology, which can make earlier presentations harder to recognize on exam [3,4]. Nevertheless, the subtle reflective surface texture combined with prominent pruritus in our patient raised clinical suspicion for underlying LS. Prior overlap reports emphasize that when depigmentation is prominent, clinicians should intentionally assess for symptom-driven and surface clues such as pruritus, fissures or erosions, purpura or ecchymoses, and subtle textural change [3]. In this setting, a low threshold for biopsy helps establish definitive clinicopathologic correlation and guide management [4].

Histologically, our biopsy demonstrated definitive LS features critical for diagnosis, notably dermal hyalinization and a dense, band-like lymphocytic infiltrate [2]. Importantly, the complete absence of melanocytes confirmed by Melan-A immunostaining strongly supported a concurrent diagnosis of vitiligo rather than pigmentary changes secondary solely to inflammation [1]. Because LS-associated leukoderma can occur through multiple mechanisms, including variable degrees of melanocyte loss, the clinical and histologic boundary with vitiligo may be blurred when depigmentation dominates the presentation [5]. Carlson et al. reported that LS has a lower mean melanocyte count than controls and that a minority of LS cases can show melanocyte loss comparable to vitiligo, demonstrating that melanocyte depletion can occur in LS [5]. Consistent with this, Park et al. showed significant variability in melanocyte numbers within LS lesions, supporting careful immunohistochemical evaluation when distinguishing concurrent vitiligo from LS-associated hypopigmentation [6]. Accordingly, in our case, LS-defining histology together with the complete absence of Melan-A-positive melanocytes supported true concurrent vitiligo rather than LS-related hypopigmentation alone when interpreted in a clinicopathologic context [5,6].

Although the precise autoimmune relationship between LS and vitiligo remains unclear, Weisberg et al. noted “epitope spreading” as a potential explanatory mechanism, suggesting that initial tissue damage from one autoimmune disorder could expose previously hidden melanocytic antigens, thus triggering a subsequent autoimmune response directed against melanocytes [7]. Carlson et al. similarly hypothesized that lichenoid inflammation in LS may provoke an autoimmune reaction targeting melanocytes, leading to melanocyte-directed autoimmunity [5]. These proposed mechanisms remain hypothetical and do not establish a causal relationship, but in our case, the co-localization of LS-associated lichenoid inflammation with complete melanocyte loss is compatible with these hypotheses [5,7]. Despite these shared autoimmune features and pathogenetic hypotheses, the conditions remain distinct clinically and histologically.

Accurate differentiation remains crucial due to clinically distinct therapeutic approaches. LS treatment utilizes high-potency topical steroids to target suppression of chronic inflammation to prevent tissue scarring, anatomical distortion, and potential malignant transformation [2]. Conversely, vitiligo therapy prioritizes repigmentation, frequently employing adjunctive treatments such as phototherapy, topical calcineurin inhibitors, and corticosteroids [1]. In overlap presentations, this distinction has direct management implications because depigmentation can obscure clinically active LS that still requires treatment [3]. Misdiagnosis or delayed identification can postpone appropriate care.

Recognizing concurrent LS and vitiligo is particularly challenging in patients with skin of color, as prominent depigmentation can obscure subtle LS features. Desai et al. introduced the term “*vitiligoid vulvar* lichen sclerosus” (VVLS) to describe LS presenting predominantly as depigmented patches resembling vulvar vitiligo, commonly observed in darker-skinned individuals. VVLS is frequently misdiagnosed due to subtle clinical features, leading to delays in appropriate treatment and subsequent increased risk of architectural distortion and malignancy [8]. Our patient parallels this VVLS pattern in that depigmentation dominated the exam while symptoms and subtle surface change were the key prompts to pursue biopsy and confirm concomitant LS [8]. Early identification of subtle LS lesions is therefore essential. Clinical vigilance is strongly recommended in ambiguous cases to ensure accurate diagnosis and timely intervention [4,8].

## Conclusions

Concurrent vulvar vitiligo and LS pose diagnostic challenges due to overlapping depigmentation and subtle clinical features. Melanocyte-specific immunostains (e.g., Melan-A) are invaluable for confirming true melanocyte loss and distinguishing vitiligo from LS-associated hypopigmentation. Clinical vigilance, a low threshold for biopsy, and targeted therapy optimize outcomes and mitigate the risks of scarring or malignancy.
